# Nummular Keratitis Following Epidemic Keratoconjunctivitis: A Guide to Managing Subepithelial Infiltrates

**DOI:** 10.7759/cureus.110958

**Published:** 2026-06-16

**Authors:** Ksheeraja Y, Madhuri P, Shilpa Anna Koshy

**Affiliations:** 1 Ophthalmology, M.S. Ramaiah Medical College and Hospital, Bangalore, IND

**Keywords:** cyclosporin, epidemic keratoconjunctivitis, nummular keratitis, topical corticosteroids, viral keratoconjunctivitis

## Abstract

Introduction: Epidemic keratoconjunctivitis (EKC) is a highly contagious viral ocular surface infection characterized by conjunctivitis with corneal involvement. Although EKC is usually self-limiting, corneal sequelae can persist for weeks to months and may significantly impact the quality of life.

Aim: To analyze the effectiveness of the treatment protocol and healing of nummular keratitis after EKC.

Methods: This is a retrospective observational study of eight patients with a history of viral conjunctivitis and EKC. Diagnosis and management in the acute stage relied solely on clinical evaluation, as laboratory testing remains significantly limited, often resulting in underdiagnosis and inadequate treatment. Thorough slit lamp evaluation, fluorescein staining, and visual acuity assessment were performed for all patients during follow-up. These cases of EKC were initially managed suboptimally elsewhere and later presented with decreased vision, photophobia, and nummular subepithelial infiltrates.

Results: All patients were treated with a tapering course of low-dose topical corticosteroids, leading to complete resolution in six cases (75%), while two (25%) required long-term topical cyclosporine therapy.

Conclusion: This study highlights the importance of close monitoring of patients with acute infectious conjunctivitis until full resolution, enabling timely initiation of topical immunosuppressive therapy when indicated for nummular subepithelial infiltrates. Our study suggests that treatment and follow-up protocols, along with screening for other household contacts with conjunctivitis, can significantly reduce the severity and duration of EKC and help preserve final visual acuity after resolution.

## Introduction

Acute infectious conjunctivitis is a contagious ocular condition with diverse etiologies, including bacteria, viruses, and parasites [1,2]. Viral agents account for nearly 80% of acute cases, with adenoviruses responsible for approximately 65-90%, manifesting primarily as epidemic keratoconjunctivitis (EKC) and pharyngoconjunctival fever (PCF) [2]. A smaller proportion of cases are attributed to acute hemorrhagic conjunctivitis (AHC), associated with enteroviruses and coxsackieviruses, and herpetic conjunctivitis, resulting from herpes simplex virus (HSV) infection [1,2].

EKC is caused by human adenovirus (HAdV), which belongs to the genus *Mastadenovirus* of the *Adenoviridae* family. Serotypes 8, 37, 53, 54, 64, and HAdV-D are primarily responsible for EKC [3,4]. Typical symptoms of EKC include watery discharge, foreign body sensation, redness, and photophobia. Clinical findings may reveal conjunctival chemosis, hyperemia, follicular conjunctivitis, eyelid edema, epithelial keratitis, and preauricular lymphadenopathy, while severe cases can present with pseudomembrane formation and symblepharon [5].

The incubation period of EKC ranges from two days to two weeks, and patients may remain contagious for up to three weeks after symptom onset [4,6]. In the acute phase, clinical signs of conjunctivitis are evident, with corneal involvement being the hallmark feature. This typically begins as punctate epithelial keratitis, which often resolves within a few days [3,6,7]. However, in approximately 60% of cases, multiple focal subepithelial infiltrates (“nummuli”) develop by 10-14 days. These lesions are generally small (0.5-1 mm), oval to round, uniform in size, and vary in number depending on severity. They are usually concentrated in the central cornea, scattered in the intermediate zone, and rarely observed near the limbus [3,7,8]. Patients may remain asymptomatic or may develop corneal aberrations and irregular astigmatism that can lead to reduced vision, glare, photophobia, and long-term visual impairment. These complications can persist for months, and in some cases, years, even after the acute infection has resolved and despite treatment [7].

Early-stage cases are often underdiagnosed and treated symptomatically with topical antibiotics and lubricants. Many patients later develop corneal subepithelial infiltrates (SEIs), and delayed eye examinations can worsen corneal involvement, leading to relapses and chronic disease.

Hence, we aimed to study the aftermath of EKC in patients who presented with corneal involvement and decreased vision post conjunctivitis, assessing their response to treatment and final visual outcomes.

## Materials and methods

This retrospective observational study was conducted at M.S. Ramaiah Medical College and Hospital, Bangalore, India, over a six‑month period from June 1, 2025, to December 1, 2025. The study included eight patients who developed subepithelial nummular infiltrates following EKC. Clinical records of all patients presenting with post‑EKC corneal infiltrates during the study period were reviewed. A pragmatic approach was adopted, utilizing the maximum number of records to provide the most robust estimates possible within the constraints of existing data. Data collected included demographic details, clinical presentation, duration of symptoms, treatment modalities, and outcomes.

This case series included patients diagnosed with EKC who subsequently developed nummular keratitis/SEIs during follow-up. Demographic information, presenting clinical findings, visual acuity, slit-lamp examination findings, treatment details, and clinical outcomes were obtained from the medical records. All patients underwent comprehensive ophthalmic evaluation, and treatment decisions were based on the severity and location of corneal infiltrates. Follow-up examinations were performed to assess resolution of infiltrates, improvement in visual acuity, and the need for additional immunomodulatory therapy.

## Results

Case 1

A 42-year-old male, who had undergone right eye cataract surgery one year ago, presented with complaints of redness, discharge, and itching in both eyes for three weeks. He had previously been treated elsewhere with ciprofloxacin eye drops, which he discontinued after one week. He presented to our OPD as his symptoms persisted, and he started experiencing blurry vision and photophobia.

**Figure 1:**
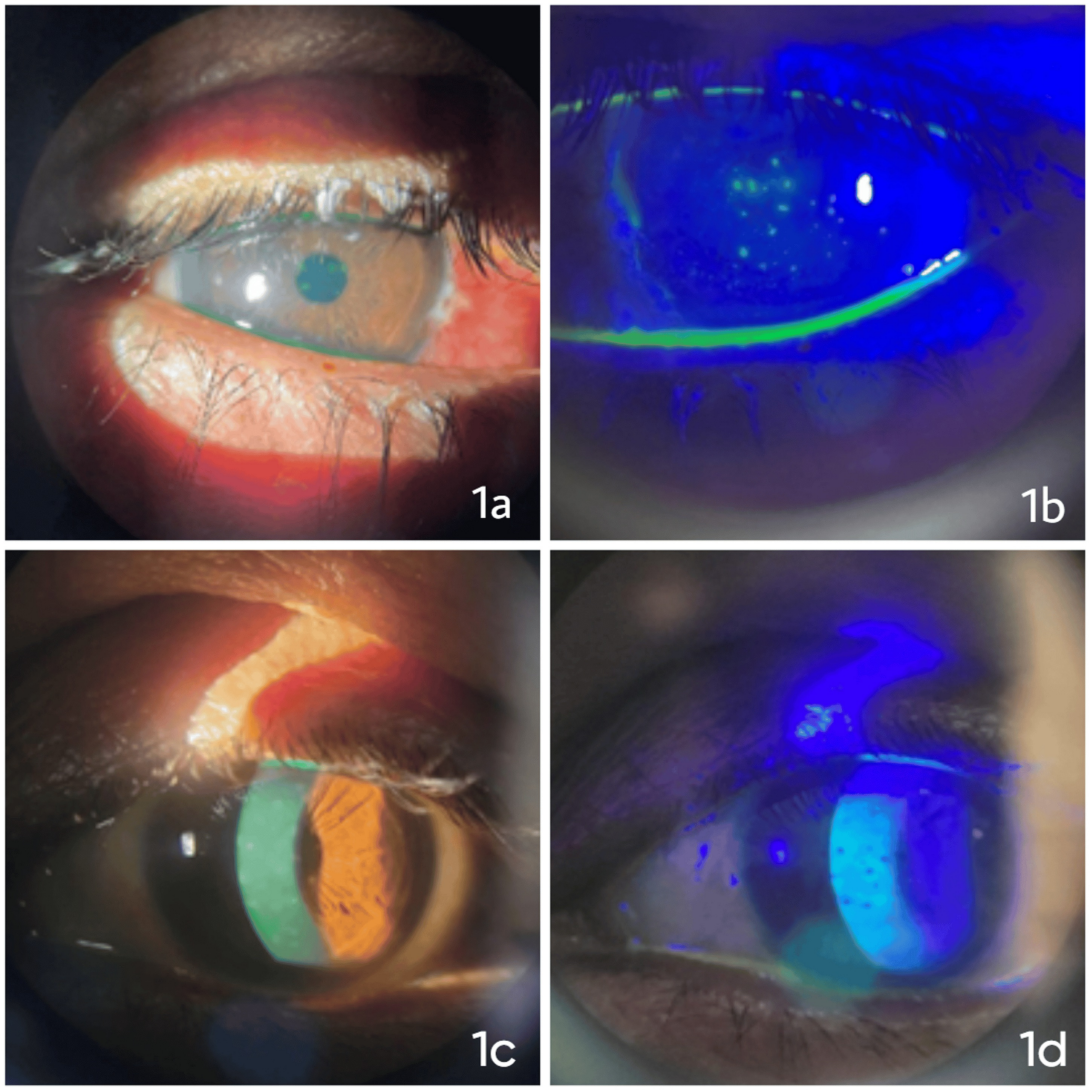
(1a) Right eye slit lamp picture showing SEIs (1b) with positive FS. (1c) Left eye slit lamp picture at presentation showing SEIs (1d) with positive FS. SEIs: subepithelial infiltrates; FS: fluorescein staining.

On examination, vision was 6/18 in both eyes. Anterior segment evaluation revealed diffusely congested conjunctiva and multiple nummular infiltrates with positive fluorescein staining concentrated over the central cornea in the right eye. The left eye showed minimal conjunctival congestion and nummular infiltrates with a cataractous lens (nuclear sclerosis grade II), as shown in Figure 1.

**Figure 2:**
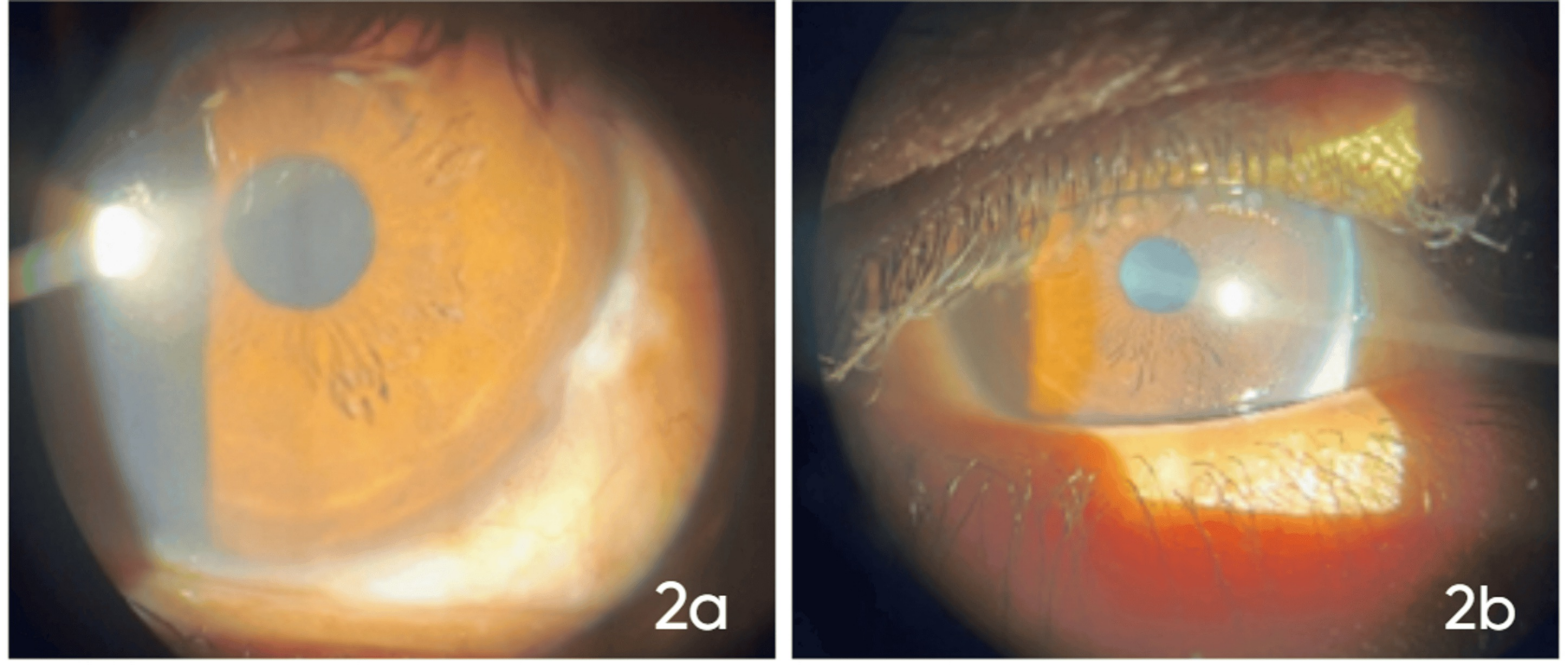
Slit lamp pictures showing resolution of subepithelial infiltrates post treatment at four weeks follow-up in the (2a) right eye and (2b) left eye.

He was started on moxifloxacin 0.5% eye drops four times a day and loteprednol etabonate 0.5% ophthalmic suspension four times a day with tapering one drop per week. The patient was closely followed up, and after two weeks of treatment, vision in the right eye improved to 6/6, and corneal infiltrates faded, leaving nummular opacities. Left eye vision improved to 6/9, and the cornea was cleared. The anterior segment picture at the four-week follow-up is shown in Figure 2. The patient was followed up for up to six months to ensure complete resolution.

Case 2

A 30-year-old male presented with complaints of redness and discharge in both eyes for one week and had been treated elsewhere with topical antibiotics. On evaluation, vision was 6/9 in both eyes, and anterior segment examination revealed diffuse conjunctival congestion with clear corneas bilaterally.

**Figure 3:**
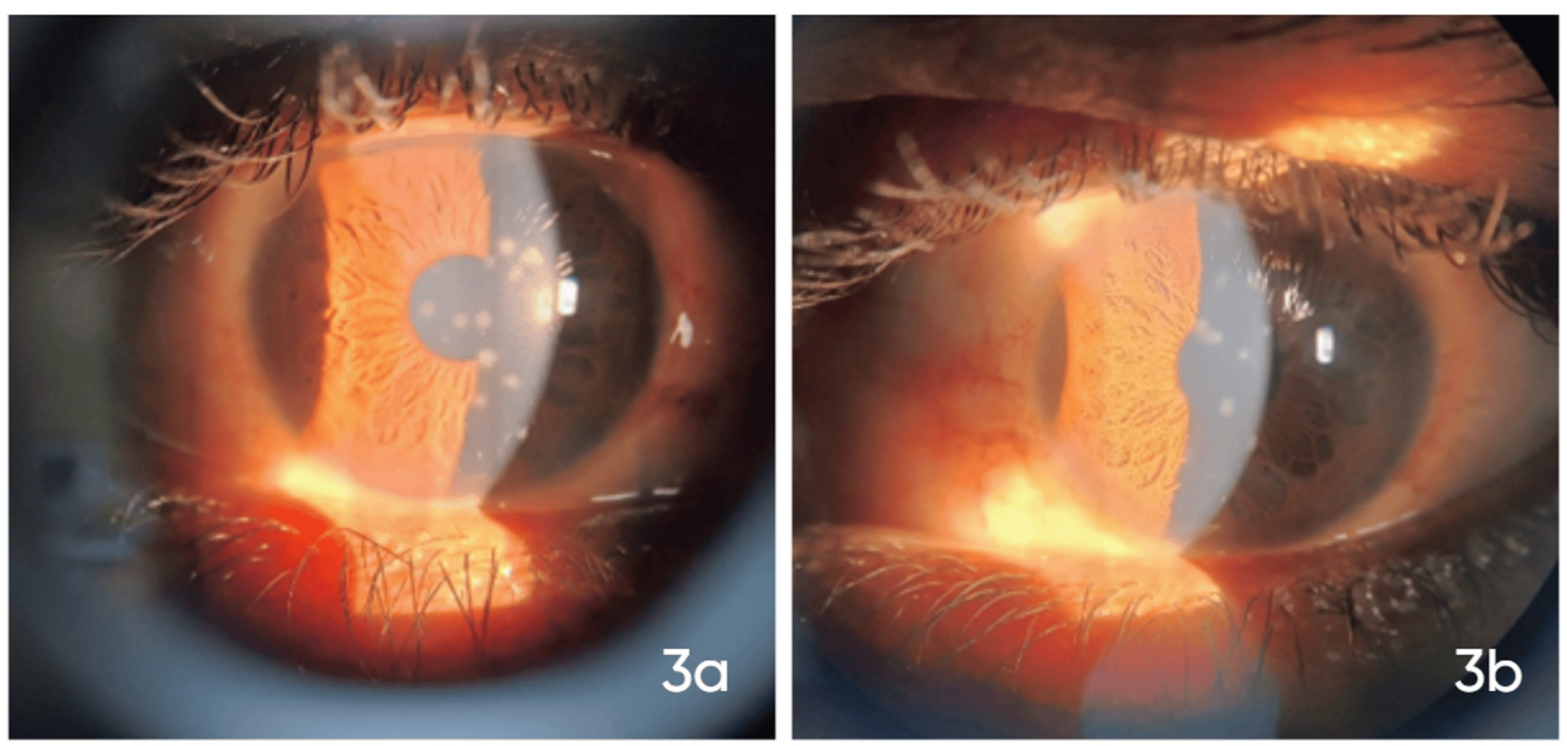
Nummular keratitis with multiple subepithelial infiltrates in the (3a) right eye and (3b) left eye.

The patient was started on moxifloxacin 0.5% eye drops eight times a day along with adequate lubricating eye drops. He was followed up closely, and at the third-day review, vision had dropped to 6/12 in the right eye and 6/18 in the left eye. Anterior segment examination showed diffuse superficial punctate keratitis with positive fluorescein staining in both eyes, which progressed to SEIs with negative fluorescein staining over the next five days, predominantly affecting the central cornea, as seen in Figure 3.

The patient was then started on loteprednol etabonate 0.5% ophthalmic suspension five times a day, with a tapering course. Final visual acuity improved at six weeks, with 6/9 in both the right and left eyes, with faded nummular opacities.

Case 3

A 56-year-old male presented with complaints of blurred vision and redness in both eyes for one week. He reported a history of eye infection two weeks prior, which had been treated elsewhere with topical antibiotics and lubricating eye drops for one week before being discontinued.

**Figure 4:**
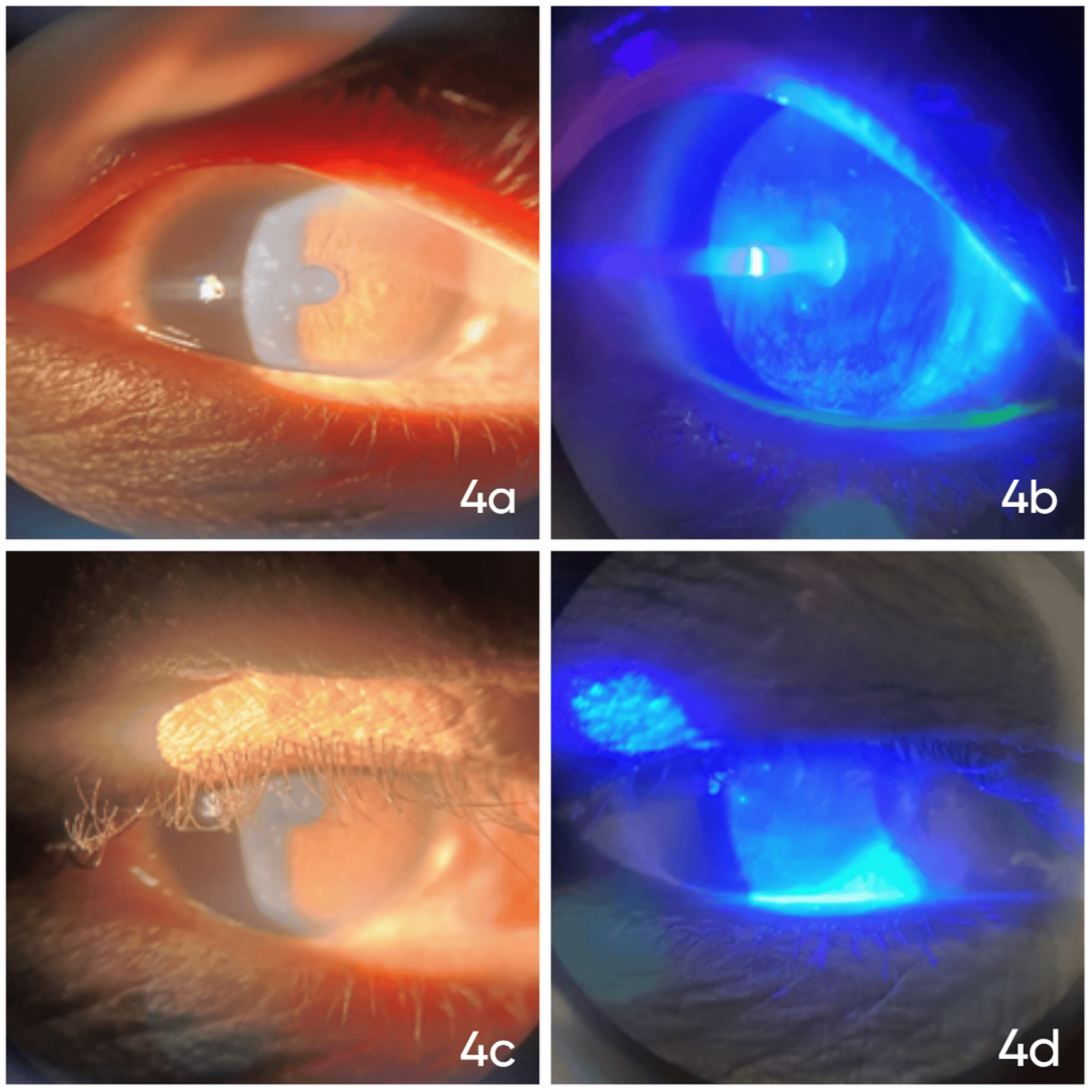
Slit lamp picture showing subepithelial infiltrates and positive fluorescein staining in the (4a, 4b) right eye and (4c, 4d) left eye.

On evaluation, vision was 6/24 in the right eye and 6/6 in the left eye. Anterior segment examination showed diffuse conjunctival congestion and corneal punctate keratitis, with multiple SEIs more concentrated in the central cornea of both eyes, as shown in Figure 4.

The patient was started on moxifloxacin 0.5% eye drops, loteprednol etabonate 0.5% ophthalmic suspension five times a day, and adequate lubricating drops and gel. Despite steroid treatment for one month, he continued to have decreased vision and photophobia. Therefore, topical cyclosporine 0.09% drops three times daily were initiated for three months. At six weeks, visual acuity had improved to 6/9 in the right eye with faded opacities and 6/6p in the left eye with complete resolution of opacities.

Cases 4, 5, and 6

**Figure 5:**
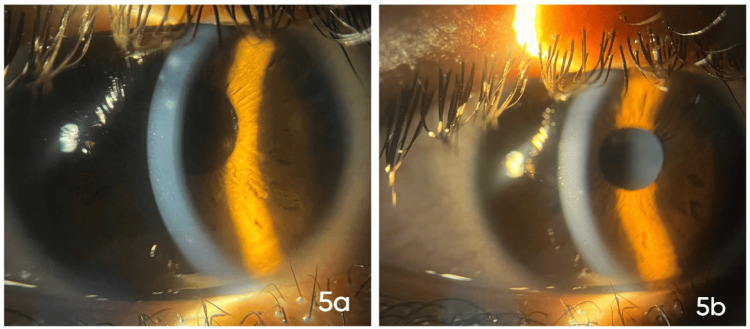
Slit lamp picture showing subepithelial infiltrates of case 4 in the (5a) right and (5b) left eye.

Three members of the same family (a mother, father, and son) had a history of conjunctivitis 10 days prior. The father, aged 53 years, presented with photophobia and decreased vision for three days, along with a history of redness for 10 days. On examination, he had multiple nummular SEIs with positive fluorescein staining in both eyes, as shown in Figure 5.

His 18-year-old son had experienced redness for two weeks and decreased vision for five days. He also had SEIs with positive fluorescein staining. The 50-year-old mother had redness for one week, with very few nummular lesions on the cornea, and was not as symptomatic as the other two.

All three patients were started on moxifloxacin 0.5% eye drops, loteprednol etabonate 0.5% ophthalmic suspension five times a day (with weekly tapering), and adequate lubricating drops and gel. The corneal lesions resolved completely in the mother and son, but the father had residual nummular lesions and photophobia. He was therefore started on topical cyclosporine 0.09% eye drops along with loteprednol for three months. At the end of three months, his lesions had resolved, and vision improved to 6/6 in the right eye and 6/6P in the left eye.

Cases 7 and 8

**Figure 6:**
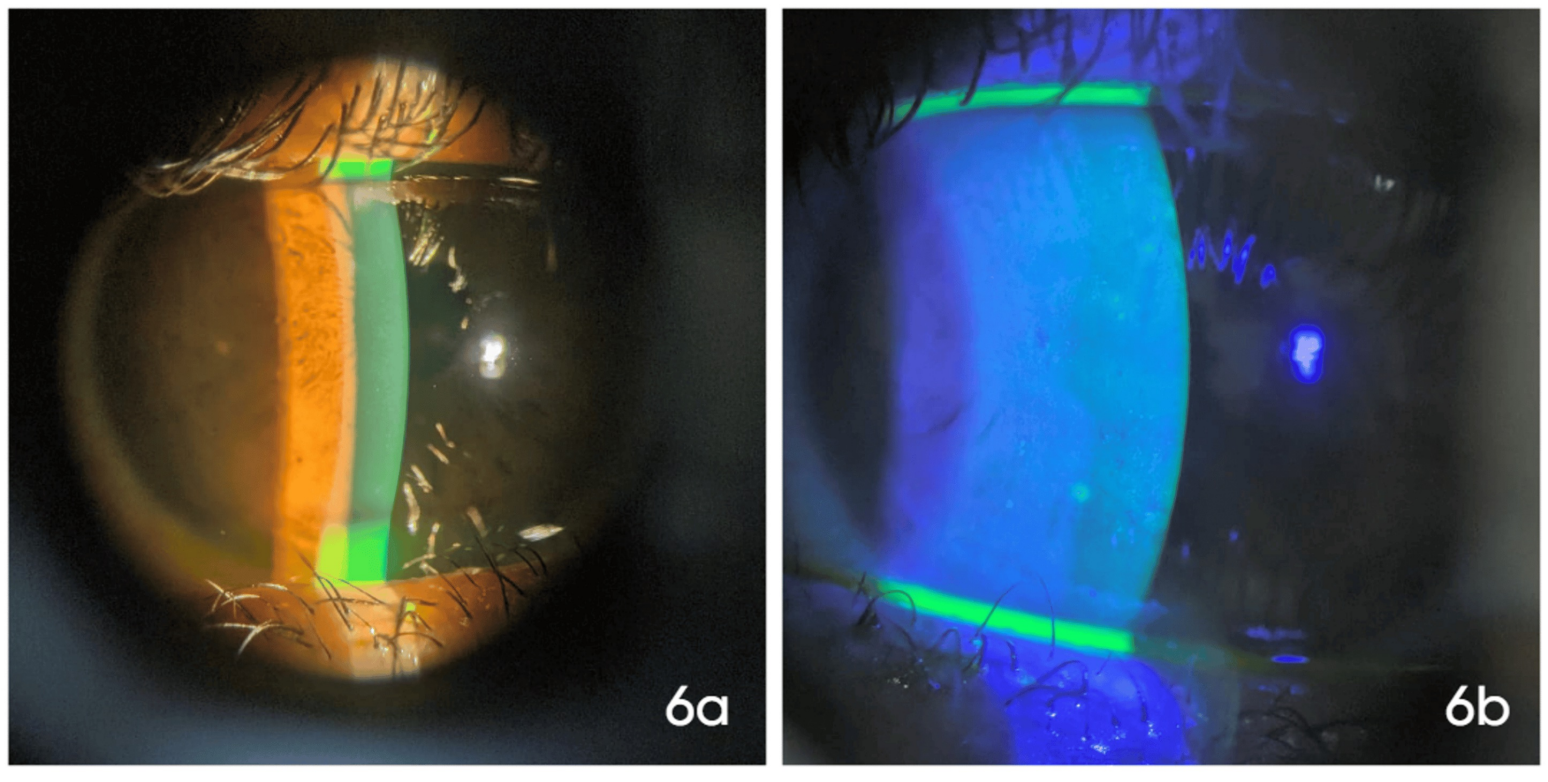
Slit lamp image of case 7 showing (6a) subepithelial infiltrates with (6b) fluorescein staining.

A husband and wife presented with a history of redness in both eyes for 10 days and blurred vision and photophobia for three days. On examination, the wife had conjunctival congestion and a few SEIs in the pupillary area, with vision of 6/12 in both eyes. The husband had a few SEIs with vision of 6/9 in both eyes. Both were treated with topical loteprednol five times a day. After six weeks, they improved significantly, achieving 6/6 vision in both eyes with complete resolution of nummular lesions, as shown in Figure 6.

Clinical presentation and treatment outcomes

Eight patients (16 eyes) with EKC-associated nummular keratitis were included in the study. Corneal involvement ranged from superficial punctate keratitis progressing to SEIs to multiple central nummular lesions, with presenting visual acuity ranging from 6/6 to 6/24. Following treatment with topical moxifloxacin and tapering loteprednol etabonate, with adjunctive topical cyclosporine in selected severe cases, complete resolution or marked fading of infiltrates was observed in all eyes, with only minimal residual scarring noted in one eye (Table 1).

**Table 1:** Clinical presentation and treatment followed for each case of EKC. EKC: epidemic keratoconjunctivitis; OD: oculus dexter; OS: oculus sinister; FS: fluorescein staining; SEIs: subepithelial infiltrates; SPK: superficial punctate keratitis; E/D: eye drops; TID: three times a day; QID: four times a day; 5T/D: five times a day.

S. No.	Vision on presentation	Corneal finding	Treatment	Post-treatment vision at 4 weeks	Corneal response to treatment
Case 1	OD - 6/18	Multiple nummular infiltrates, more at the central cornea; FS positive	· Moxifloxacin 0.5% E/D QID. Loteprednol etabonate 0.5% ophthalmic suspension QID (tapering course)	6/6	Complete resolution of nummular lesions
	OS - 6/18	Multiple SEIs, milder in comparison to the right eye	· Moxifloxacin 0.5% E/D QID. Loteprednol etabonate 0.5% ophthalmic suspension QID (tapering course)	6/9	Faded nummular opacities
Case 2	OD - 6/9. After 1 week - 6/12	3rd day after onset of symptoms- SPKs developed (FS+), which progressed to SEI in another 5 days (FS-)	· Moxifloxacin 0.5 % E/D 8T/D. Later on, progression started on loteprednol etabonate 0.5% ophthalmic suspension 5T/D (tapering course)	6/9	Faded nummular opacities
	OS - 6/9. After 1 week - 6/18	3rd day after onset of symptoms - SPKs developed (FS+), which progressed to SEI in another 5 days (FS-)	Moxifloxacin 0.5 % E/D 8T/D. Later on, progression started on loteprednol etabonate 0.5% ophthalmic suspension 5T/D (tapering course)	6/6p	Faded nummular opacities
Case 3	OD - 6/24	Multiple SEIs concentrated at central cornea	· Moxifloxacin 0.5% E/D QID, loteprednol etabonate 0.5% ophthalmic suspension 5T/D (tapering course), topical cyclosporin 0.09% TID for 3 months	6/9	Faded nummular opacities, one in the pupillary area
	OS - 6/6		· Moxifloxacin 0.5% E/D QID, loteprednol etabonate 0.5% ophthalmic suspension 5T/D (tapering course), topical cyclosporin 0.09% TID for 3 months	6/6	Complete resolution of nummular lesions
Case 4 (father)	OD - 6/18	Multiple SEIs, positive fluorescein staining in the pupillary area	· Moxifloxacin 0.5% E/D QID, loteprednol etabonate 0.5% ophthalmic suspension 5T/D (tapering course) along with topical cyclosporin 0.09% eye drops for 3 months	6/9p	Faded nummular opacities, two in the pupillary area
	OS - 6/12	Multiple SEIs	· Moxifloxacin 0.5% E/D QID, loteprednol etabonate 0.5% ophthalmic suspension 5T/D (tapering course) along with topical cyclosporin 0.09% eye drops for 3 months	6/6p	Faded nummular lesions with faint scarring
Case 5 (son)	OD - 6/9	Multiple SEIs	Moxifloxacin 0.5% E/D QID. Tapering loteprednol etabonate 0.5% ophthalmic suspension 5T/D	6/6	Complete resolution of nummular lesions
	OS - 6/9P	Multiple SEIs	Moxifloxacin 0.5% E/D QID. Tapering loteprednol etabonate 0.5% ophthalmic suspension 5T/D	6/6	Complete resolution of nummular lesions
Case 6 (mother)	OD - 6/6p	Few SEIs	Moxifloxacin 0.5% E/D QID. Tapering loteprednol etabonate 0.5% ophthalmic suspension 5T/D	6/6	Complete resolution of nummular lesions
	OS - 6/6p	Few SEIs	Moxifloxacin 0.5% E/D QID. Tapering loteprednol etabonate 0.5% ophthalmic suspension 5T/D	6/6	Complete resolution of nummular lesions
Case 7 (husband)	OD - 6/9	Few SEIs	Moxifloxacin 0.5% E/D QID. Tapering loteprednol etabonate 0.5% ophthalmic suspension 5T/D	6/6	Complete resolution of nummular lesions
	OS - 6/9	Few SEIs	Moxifloxacin 0.5% E/D QID. Tapering loteprednol etabonate 0.5% ophthalmic suspension 5T/D	6/6	Complete resolution of nummular lesions
Case 8 (wife)	OD - 6/9	Few SEIs	Moxifloxacin 0.5% E/D QID. Tapering loteprednol etabonate 0.5% ophthalmic suspension 5T/D	6/6	Complete resolution of nummular lesions
	OS - 6/9	Few SEIs	Moxifloxacin 0.5% E/D QID. Tapering loteprednol etabonate 0.5% ophthalmic suspension 5T/D	6/6	Complete resolution of nummular lesions

## Discussion

Viral conjunctivitis is a highly contagious ocular disease that imposes a significant burden on patients' quality of life and healthcare systems. Although generally self-limiting, it can disrupt daily functioning and cause considerable discomfort, particularly when complicated by sequelae such as SEIs, corneal scarring, conjunctival membrane formation, and cicatrising conjunctivitis with symblepharon [9,10]. The exact etiopathogenesis of SEIs is unclear, although it appears to be associated with the accumulation of lymphocytes and monocytes/macrophages recruited in response to fibroblasts activated by HAdV infection and capable of producing proinflammatory mediators, such as chemokines, including interleukin-8 [7]. Despite the detrimental effect that adenoviral (mainly HAdV) infection poses, there is currently no FDA-approved drug to treat these conditions, making management difficult [10].

The role of mild topical corticosteroids with adequate tapering doses has been studied previously. Their use can effectively relieve EKC symptoms without significant adverse effects when used briefly in the acute phase, and they also help manage persistent SEIs in the post-acute stage [10].

A study conducted by Koçluk et al. comparing the efficacy and potential ocular side effects of dexamethasone and loteprednol eyedrops for SEIs concluded that both drugs have similar efficacy, with loteprednol causing fewer ocular side effects [11].

Another study by Gouider et al. found that topical cyclosporine A 0.5% and fluorometholone worked favorably for SEIs, and both are safe options [12].

Our case series highlights the clinical significance of corneal SEIs following viral conjunctivitis and underscores the importance of timely intervention and long-term follow-up. We treated patients with corneal SEI using loteprednol 0.5% ophthalmic suspension with adequate tapering dosage. The clinical presentation and treatments followed are summarized in Table 1. Most patients in this series were initially treated inadequately and later presented with complaints of blurred vision and photophobia. All patients improved both symptomatically and clinically. One patient did not respond well to topical low-dose steroids and was therefore started on cyclosporine A 0.5% eye drops for six months. All patients were followed up at regular intervals, depending on clinical presentation, for a duration of six months to one year until complete resolution was achieved.

Limitations of our study included a relatively small number of patients, which limits the generalizability of the findings. Hence, further larger, randomized studies comparing corticosteroids, cyclosporine A, and other immunomodulators are needed to establish optimal treatment protocols.

## Conclusions

This study reinforces the necessity of vigilant follow-up in viral conjunctivitis cases for at least four to six months, enabling early detection of SEIs and initiation of anti-inflammatory therapy. Such an approach not only improves patient outcomes but also reduces the risk of long-term sequelae, such as corneal scarring and visual impairment. Our study emphasizes that not every red eye is treated with only antibiotics. A thorough slit lamp examination with the ophthalmologist is required in all viral conjunctivitis patients, not only in the acute stage but also up to a month, to look for corneal staining and nummular SEIs, if any. Only some patients with central lesions present to us with decreased vision. Screening all household contacts of patients with conjunctivitis is mandatory if they also have a red eye. As the timing of nummular SEI development varies among individuals, regular follow-up and patient education regarding treatment adherence are essential to facilitate early symptom resolution and achieve favorable final visual outcomes. Effective treatment protocol for EKC with nummular keratitis is topical steroid therapy four times a day, with a tapering dose of one month to six weeks based on the resolution time of the lesions. If the lesions do not completely resolve with steroids, topical cyclosporine eye drops may be administered three times daily for three months, resulting in complete lesion resolution and favorable final visual acuity.
